# Single Prazosin Infusion in Prelimbic Cortex Fosters Extinction of Amphetamine-Induced Conditioned Place Preference

**DOI:** 10.3389/fphar.2017.00530

**Published:** 2017-08-10

**Authors:** Emanuele C. Latagliata, Luisa Lo Iacono, Giulia Chiacchierini, Marco Sancandi, Alessandro Rava, Valeria Oliva, Stefano Puglisi-Allegra

**Affiliations:** ^1^Fondazione Santa Lucia IRCCS Rome, Italy; ^2^Dipartimento di Psicologia, Sapienza Università di Roma Rome, Italy; ^3^Dipartimento di Biologia e Biotecnologie “Charles Darwin”, Sapienza Università di Roma Rome, Italy

**Keywords:** α1-adrenergic receptors, extinction, prelimbic cortex, conditioned place preference, BDNF, PSD-95, nucleus accumbens

## Abstract

Exposure to drug-associated cues to induce extinction is a useful strategy to contrast cue-induced drug seeking. Norepinephrine (NE) transmission in medial prefrontal cortex has a role in the acquisition and extinction of conditioned place preference induced by amphetamine. We have reported recently that NE in prelimbic cortex delays extinction of amphetamine-induced conditioned place preference (CPP). A potential involvement of α1-adrenergic receptors in the extinction of appetitive conditioned response has been also suggested, although their role in prelimbic cortex has not been yet fully investigated. Here, we investigated the effects of the α1-adrenergic receptor antagonist prazosin infusion in the prelimbic cortex of C57BL/6J mice on expression and extinction of amphetamine-induced CPP. Acute prelimbic prazosin did not affect expression of amphetamine-induced CPP on the day of infusion, while in subsequent days it produced a clear-cut advance of extinction of preference for the compartment previously paired with amphetamine (Conditioned stimulus, CS). Moreover, prazosin-treated mice that had extinguished CS preference showed increased mRNA expression of brain-derived neurotrophic factor (*BDNF*) and post-synaptic density 95 (*PSD-95*) in the nucleus accumbens shell or core, respectively, thus suggesting that prelimbic α1-adrenergic receptor blockade triggers neural adaptations in subcortical areas that could contribute to the extinction of cue-induced drug-seeking behavior. These results show that the pharmacological blockade of α1-adrenergic receptors in prelimbic cortex by a single infusion is able to induce extinction of amphetamine-induced CPP long before control (vehicle) animals, an effect depending on contingent exposure to retrieval, since if infused far from or after reactivation it did not affect preference. Moreover, they suggest strongly that the behavioral effects depend on post-treatment neuroplasticity changes in corticolimbic network, triggered by a possible “priming” effect of prazosin, and point to a potential therapeutic power of the antagonist for maladaptive memories.

## Introduction

Persistent memories about biologically relevant stimuli are essentials for organism and species survival. However, in some pathological conditions, highly salient memories are experienced intrusively leading the individual to maladaptive behavior (McGaugh, 2006; Sun et al., 2011; Puglisi-Allegra and Ventura, 2012). A typical example is drug addiction, a pathological condition, in which environmental stimuli or discrete cue paired with drug effects acquire the ability to induce intense drug desire that leads to drug-seeking and drug-taking (Stewart et al., 1984; Robinson and Berridge, 1993; Childress et al., 1999; Everitt et al., 2001; Shalev et al., 2002). A method to reduce this kind of responses is the extinction learning, a protocol in which repeated cues or context re-exposure in absence of the predicted event results in a decrease in magnitude and frequency of conditioned response (CR) (Myers and Davis, 2004; Myers and Carlezon, 2012). This protocol, known as exposure therapy, has been applied successfully in fear and anxiety disorders treatment. However, its application on drug addicts showed only limited success (Conklin and Tiffany, 2002). Therefore, a better understanding of neurobiological mechanisms of extinction could be important to improve the effectiveness of extinction-like protocols, and to envisage neural targets to develop pharmacological therapy.

Modulation of the conditioned stimulus (CS) motivational properties favors disengagement from drug-related cues. Thus, therapy strategies aimed at reducing the motivational properties of drug cues have been considered highly promising for successful treatment of craving and relapse in addicts (Taylor et al., 2009). In this framework, norepinephrine (NE) transmission in prefrontal cortex acquires a pivotal role to favor disengagement from drug-related cues.

Indeed, NE transmission in mPFC modulates central and behavioral responses induced by relevant biological stimuli or by neutral stimuli associated with them (Darracq et al., 1998; Feenstra et al., 2001; Mingote et al., 2004; Ventura et al., 2005, 2007, 2008; Pascucci et al., 2007; Puglisi-Allegra and Ventura, 2012).

Drugs of abuse, natural reward and aversive stimuli promotes NE increase in mPFC (Florin et al., 1994; Feenstra et al., 2001; Mingote et al., 2004; Ventura et al., 2005, 2007; Pascucci et al., 2007), leading to dopamine (DA) release in the NAc that is critical for the attribution of motivational salience to highly salient stimuli (Darracq et al., 1998; Ventura et al., 2003, 2005, 2007; Puglisi-Allegra and Ventura, 2012). Furthermore, highly salient unconditioned stimulus (US) and conditioned stimulus (CS) paired with them, increase NE levels in mPFC proportionally to the salience of the US (Feenstra et al., 2001; Mingote et al., 2004; Ventura et al., 2008). This indicates that prefrontal NE transmission modulates motivational properties of conditioned cues during exposure to them, strengthening them and contributing to the maintenance of the CR. Consistent with this view, we have recently reported that selective NE depletion in pre-limbic cortex (PL), after acquisition of amphetamine CPP, facilitates extinction of drug-associated memory (Latagliata et al., 2016).

Alpha1-adrenergic receptors (α1-ARs) in mPFC have been related to the modulation of motivational properties of salient experiences. Indeed, α1-ARs that are preferentially engaged during conditions of sustained prefrontal NE release (Ramos and Arnsten, 2007) like highly salient experiences (Mingote et al., 2004; Ventura et al., 2008), modulate both motivated behavior and dopaminergic response in NAc induced by salient stimuli (Blanc et al., 1994; Darracq et al., 1998; NicNiocaill and Gratton, 2007; Schmidt et al., 2017).

Thus, in the present work we investigated if α1-ARs in PL cortex are involved in extinction of amphetamine-induced CPP in mice.

Note that the selective α1-ARs antagonist prazosin is used in clinical set for treatment of alcohol abuse and for post-traumatic stress disorder (Raskind et al., 2003; Rasmussen et al., 2009). Moreover, one of the pioneering studies by the Collège de France team (Blanc et al., 1994) showed that infusion of prazosin in the mpFC was able to control DA functioning in the NAc along more than 1 day after treatment. This report encouraged us to use prazosin in our experimental conditions in order to assess the effects of a single treatment in animal exposed to extinction training to possibly benefit from long-lasting pharmacological effects of the compound.

Thus, in the present work we investigated the effects of prazosin infusion in the PL of C57BL/6J mice on expression and extinction of amphetamine-induced CPP, hypothesizing that a single intra-PL prazosin infusion, before CPP trial (reactivation), could reduce the motivational properties of the amphetamine paired CS, weakening the persistence of amphetamine-induced CPP.

In a first set of experiments, we observed that intra-PL prazosin did not affect expression of amphetamine-induced CPP the day of infusion, while in subsequent days, when animals were drug free, it produced an early extinction in comparison with vehicle infused group. This suggested that PL α1-ARs blockade could have induced neuroplastic adaptations in mesocorticolimbic areas of prefrontal-accumbal network known to modulate incentive salience and extinction.

To assess this hypothesis, in a second set of experiments, we assessed the transcriptional modulation of BDNF and PSD-95 genes as markers of neuroplasticity and synaptic maturation, respectively, in PL and infralimbic cortex (IL), nucleus accumbens (NAc) shell and core, of prazosin treated mice having extinguished preference for amphetamine CS.

## Materials and Methods

### Animals

Male C57BL/6JIco (Charles River, Como, Italy) were purchased at 6–7 weeks of age and housed four per cage on a 12-h light–dark cycle (lights on between 07.00 a.m. and 07.00 p.m.) for 3 weeks. Two days before experiments, animals were individually housed. Each experimental group consisted of 7–10 animals. All experiments were carried out in accordance with Italian national law (DL 116/92 and DL 26/2014) on the use of animals and with the European Communities Council Directives (86/609/EEC and 2010/63/UE), and approved by the ethics committee of the Italian Ministry of Health (license/approval ID #: 10/2011-B and 42/2015-PR).

### Drugs

D-Amphetamine sulfate (Amph) and Prazosin hydrochloride (prazosin), were purchased from Sigma (Sigma Aldrich, Milan, Italy). Fluorescently labeled prazosin, BODYPY FL, was purchased by Thermo Fisher Scientific, Italy. Amph (2.5 mg/Kg), was dissolved in saline (0.9% NaCl) and injected intraperitoneally (i.p.) in a volume of 10 ml/kg. Zoletil 100, Virbac, Milan, Italy (tiletamine HCl 50 mg/ml + zolazepam HCl 50 mg/ml) and Rompun 20, Bayer S.p.A Milano, Italy (xylazine 20 mg/ml), purchased commercially, were used as anesthetics. Prazosin and BODYPY FL (1mg/ml) were dissolved in artificial CSF (in mM: NaCl 140.0; KCl 4.0; CaCl_2_ 1.2; MgCl_2_ 1.0). Artificial CSF was used as Vehicle. The doses of Amph and prazosin were selected on the bases of previous studies (Darracq et al., 1998; Do-Monte et al., 2013; Latagliata et al., 2016) and preliminary experiments.

### Apparatus

A CPP apparatus (Cabib et al., 1996, 2000) was used for behavioral experiments. The apparatus comprised two gray Plexiglas chambers (15 cm × 15 cm × 20 cm) and a central alley (15 cm × 5 cm × 20 cm). Two sliding doors (4 cm × 4 cm) connected the alley to the chambers. In each chamber, two triangular parallelepipeds (5 cm × 5 cm × 20 cm) made of black Plexiglas and arranged in different patterns (always covering the same surface of the chamber) were used as conditioned stimuli. Behavioral data were registered and analyzed by a fully automated video tracking system (“EthoVision”, Noldus Information Technology, Wageningen, The Netherlands). The acquired digital signal was processed by the software, to extract the “time spent” [in second (s)] in the three chambers of the apparatus.

### Experimental Procedures

**FIGURE 1:**
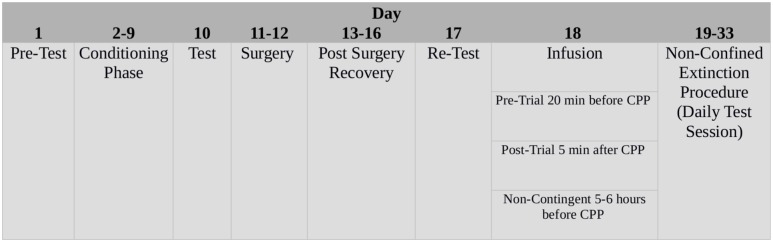
Schematic time-line of the experimental procedures.

Experimental procedures are summarized in **Figure 1**.

### Conditioned Place Preference

The training procedure was described previously (Cabib et al., 1996). Briefly, on day 1 (pre-test), mice were free to explore the entire apparatus for 20 min. On the following 8 days (con-ditioning phase), mice were injected and confined daily for 40 min alternatively in one of the two chambers. One of the patterns was consistently paired with a vehicle injection and the other one with Amph injection. In order to balance the pairings for half of the animals in each experimental group Amph (or vehicle) was paired with one of the patterns and half of them with the other one. Testing was carried out on day 10 in drug-free state and lasted 20 min like the pre-test. Note that during pre-test, mice randomly assigned to vehicle (*n* = 10) or prazosin (*n* = 10) groups did not show preference (mean time spent ± SEM), for the lateral chambers, thus showing that the apparatus was unbiased in terms of preferences in untreated mice.

### Surgery, Re-test and Infusions

The days following CPP test (days 11 and 12), animals were subjected to surgical procedures. Mice, anesthetized with Zoletil 100 and Rompun 20, were mounted in a stereotaxic frame (David Kopf Instruments, Tujunga, CA, United States) equipped with a mouse adapter. An incision was performed along the midline of the skull, then two holes were pierced in correspondence of the PL cortex, coordinates: AP +2.8; ML ± 0.4 DV -0.4 from the bregma, according to the atlas of Paxinos and Franklin (2001). Two steel cannulas were implanted (length: 7 mm; outer diameter: 0.7 mm, internal diameter 0.35 mm), secured with dental cement with the addition of epoxy glue.

One week after CPP test, the animals were subjected to a further test (re-test) to check that surgeries did not impair place preference. The day following CPP re-rest, bilateral injections of prazosin or vehicle (0.6 μg/0.6 μl side) were performed into PL through a stainless steel cannula (length 8.1 mm, 0.15 mm outer diameter, UNIMED, Swiss), connected to a 10 μl Hamilton syringe by a polyethylene tube and driven by a CMA/100 pump (flow rate 0.6 μl/min). After the end of the infusion the cannula was left in place for additional 30 s. Vehicle or prazosin pre-trial group received the infusion 20 min before the CPP trial. Post trial groups (vehicle or prazosin) received injection in PL 5 min after CPP trial. Non-contingent groups received PL infusion of vehicle or prazosin 5–6 h before the CPP trial.

### Placement Assessment

**FIGURE 2:**
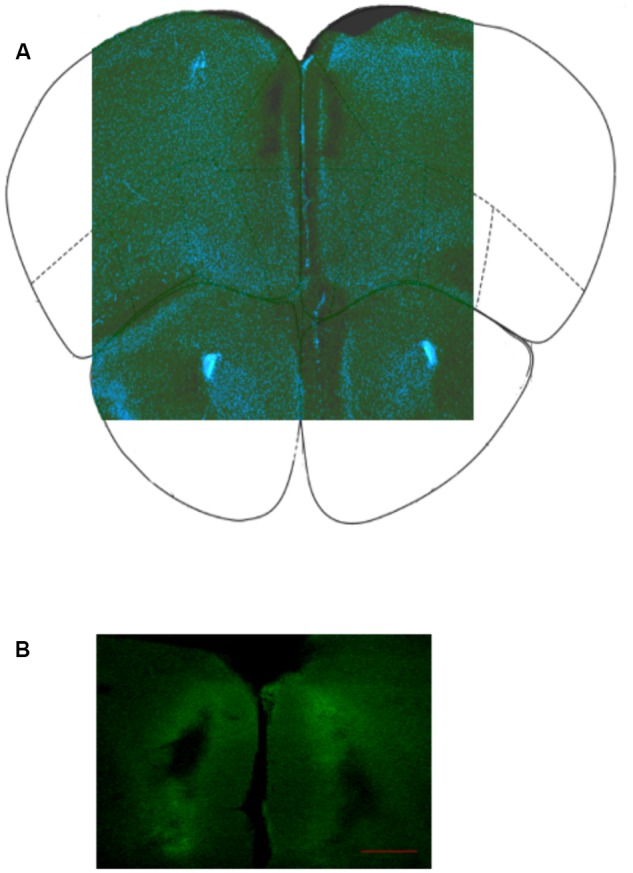
**(A)** Representative image of infusion site in PL cortex; **(B)** Location of bilateral cannula insertion to infuse fluorescently labeled prazosin (BODYPY FL) in the prelimbic cortex, PL (see Materials and Methods). The image is representative coronal photomicrographs of PL. The fluorescent trace was used to evaluate the selectivity of the diffusion gradient. The clearest part indicates the maximum spread of prazosin in the PL around the cannula track. Scale bar = 200 μm.

To assess placement, drug dispersion, and tissue damage in the PL a solution of a CSF containing the fluorescently labeled BODYPY FL prazosin was infused as described before. **Figure 2** shows a representative image of the preparation used to determine location of the cannula in the PL after infusion with CSF (**Figure 2A**) or fluorescently labeled prazosin (**Figure 2B**).

Placements in PL were judged by methylene blue staining. Brains were post-fixed in 4% paraformaldehyde, cut in serial coronal slices according to Paxinos and Franklin (2001) and processed for methylene blue staining. In order to reconstruct the correct cannula placements 50-μm thick sections were examined under a microscope to establish the location of the cannula. In **Figure 2** is represented the location of cannulas in the two hemispheres. Data from animals not showing the proper placement (*n* = 9 for all experiments) were discarded from the final statistical analysis.

### Extinction

The extinction procedure began the day after the re-test. To investigate potential time-dependent differences in the extinction, animals were exposed daily to CPP test (20 min) (Orsini et al., 2008) (non-confined extinction). The extinction of the CR was considered acquired after two consecutive days showing non-significant preference for the drug-paired chamber (Fricks-Gleason and Marshall, 2008; Latagliata et al., 2016).

### Quantitative Real Time RT-PCR and Gene Expression Analysis

After extinction of prazosin treated mice, the animals of the four experimental groups were sacrificed, brains were removed and stored in liquid nitrogen. Then, after brains were fixed vertically on the freeze plate of a freezing microtome, punches of both hemispheres were obtained from the brain slices (coronal sections) no thicker than 300 μm. Stainless steel tubes of 1.0 mm of inside diameter for PL and 0.5 mm for IL, NAc Core and NAc Shell were used. The coordinates were measured according to the Paxinos and Franklin (2001) atlas (coronal sections as mm from bregma), as follows: PL two slices from 2.80 to 2.22; IL two slices from 2.10 to 1.54; NAc Core and Shell three slices from 1.88 to 0.98. The punches were stored in liquid nitrogen until the day of RNA extraction.

RNA was isolated from brain punches using Total RNA purification Kit (Norgen Biotek, Thorold, ON, Canada) according to the manufacturer protocol. RNA quantity was determined by absorbance at 260 nm using a NanoDrop UV-VIS spectrophotometer. Complementary DNA was obtained using the High Capacity Reverse Transcription Kit (Applied Biosystems, Branchburg, NJ, United States). cDNA templates (8 ng) were amplified with quantitative PCR using the Taqman technology in the 7900HT thermal cycler apparatus equipped with the SDS software version 2.3 (Applied Biosystems) for data collection. Taqman primer sets (Applied Biosystems) were used to amplify mouse total *BDNF* (Mm04230607_s1; amplifying the coding region for mature BDNF) and Disks large homolog 4 (Dlg4 Mm00492193_m1), gene encoding for *PSD-95*. Ct values were normalized to measures of Glyceraldehyde 3-phosphate dehydrogenase (GAPDH Mm99999915_g1) mRNA. All data were run in triplicate and analyzed using the ΔΔC(t) method (Schmittgen and Livak, 2008). Results are expressed as fold changes relative to the correspondent vehicle-treated group.

### Statistics

Time spent (s) in each of the three chambers was used as a dependent measure. Data were analyzed by repeated-measures ANOVA with one between factor (treatment, two levels: vehicle, prazosin) and one within factor (choice, three levels: center, paired, and unpaired). *Post hoc* comparisons were assessed by Duncan’s multiple-range test whenever significant main effects were attained. A significant CPP was indicated by a significant difference between time spent in paired versus unpaired chamber.

*BDNF* and *PSD-95* mRNA levels of expression were compared between prazosin and vehicle group by paired *t*-test with significant values attributed when *p* < 0.05.

## Results

### Prelimbic Prazosin Infusion Fosters Extinction of Conditioned Place Preference Induced by Amphetamine

We first investigated the effects of PL α1-ARs antagonist infusion on expression and extinction of Amph induced CPP. Two-way ANOVA showed non-significant (ns) effect for treatment × choice interaction: *F*(2,36) = 2.668, ns and for choice: *F*(2,36) = 1.932, ns.

**FIGURE 3:**
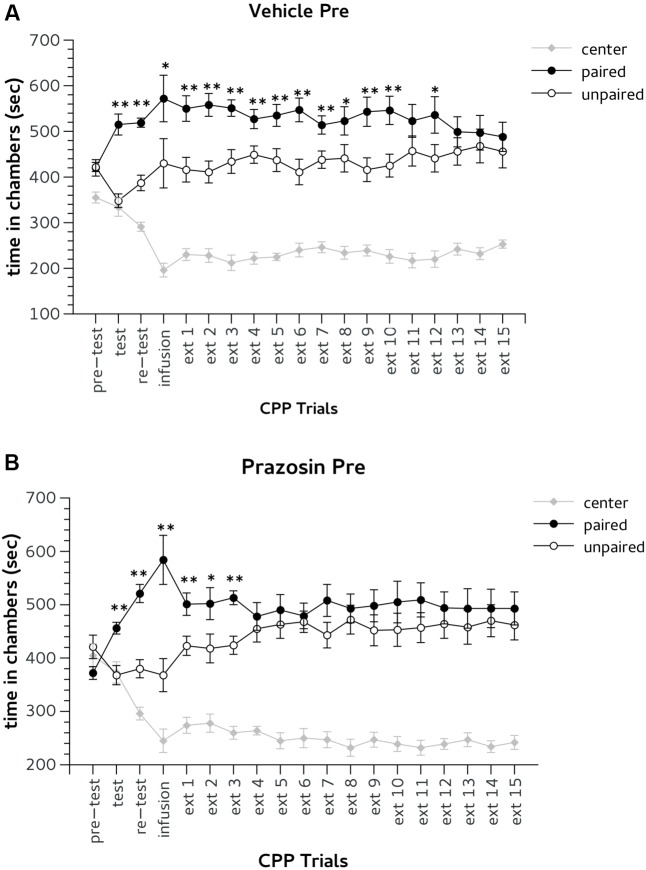
Effects of pre-trial infusion of prazosin in prelimbic (PL) prefrontal cortex on expression and extinction of an acquired conditioned place preference (CPP) induced by systemic injection of 2.5 mg/Kg of amphetamine. In the *x*-axis are days. In the *y*-axis is time spent in center, paired, and unpaired chamber during pre-test, test, re-test, and non-confined extinction trials in animals assigned to vehicle **(A)** and prazosin **(B)**. All data are expressed as mean (second ± SE) time spent in center, paired, and unpaired chambers. ^∗^*p* < 0.05, ^∗∗^*p* < 0.01 in time spent in paired in comparison with unpaired chamber in vehicle and prazosin infused mice.

Following conditioning (test) and in the trial after surgery (re-test), all mice expressed a preference for the previously Amph-paired chamber (**Figures 3A,B**). Two-way ANOVA revealed a significant effect for factor choice: [test, *F*(2,36) = 20.865, *p* < 0.01; re-test, *F*(2,36) = 80.789, *p* < 0.01]. Duncan’s *post hoc* analysis showed that both vehicle and prazosin animals spent more time in the Amph-paired chamber during test and re-test (*p* < 0.01) (**Figures 3A,B**).

Bilateral infusion of both prazosin and vehicle performed 20 min before the third CPP trial (infusion, **Figure 1**) did not affect the expression of Amph CPP (**Figures 3A,B**). Two-way ANOVA showed a significant effect for choice: [infusion, *F*(2,36) = 26.705; *p* < 0.01] and *post hoc* analysis confirmed that both groups spent more time in Amph-paired chamber during this trial (prazosin *p* < 0.01; vehicle *p* < 0.05) (**Figures 3A,B**). On extinction trial 5, mice infused with prazosin reached extinction criterion of two consecutive days showing non-significant preference for the drug-paired chamber (**Figure 3B**), whereas vehicle treated reached extinction on trial 14 (**Figure 3A**). On extinction trials 4 and 5, respectively, statistical analyses revealed a significant effect of choice [*F*(2,36) = 62.186; *p* < 0.01] and [*F*(2,36) = 54.946; *p* < 0.01]. *Post hoc* test showed that on both days only vehicle spent more time in the Amph- than in the saline-paired chamber (*p* < 0.01) (**Figure 3A**).

Recently, it has been shown that PL prazosin infusion can impair fear memory re-consolidation leading to attenuation of fear responses (Do-Monte et al., 2013). To evaluate a potential re-consolidation effect in our results we performed a second experiment in which prazosin was administrated in PL cortex 5 min after CPP trial (see **Figure 1**).

As in the previous experiment, during pre-test mice randomly assigned to vehicle post trial (*n* = 7) or prazosin post trial (*n* = 7) groups did not show preference for the lateral chambers. Two-way ANOVA revealed non-significant effect for interaction group × choice: *F*(2,24) = 0.10, ns and for factor choice: *F*(2,24) = 12.994, ns.

**FIGURE 4:**
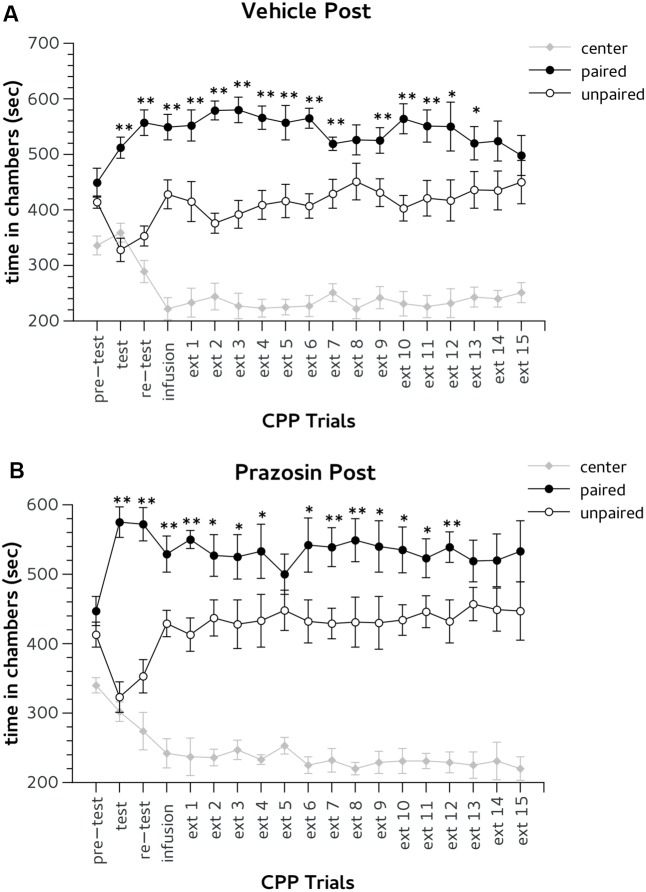
Effects of post-trial infusion of prazosin in prelimbic (PL) prefrontal cortex on expression and extinction of an acquired conditioned place preference (CPP) induced by systemic injection of 2.5 mg/Kg of amphetamine. In the *x*-axis are days. In the *y*-axis is time spent in center, paired, and unpaired chamber during pre-test, test, re-test, and non-confined extinction trials in animals assigned to vehicle **(A)** and prazosin **(B)**. All data are expressed as mean (second ± SE) time spent in center, paired, and unpaired chambers. ^∗^*p* < 0.05, ^∗∗^*p* < 0.01 in time spent in paired in comparison with unpaired chamber in vehicle and prazosin infused mice.

In both CPP test and re-test all mice showed the preference for Amph paired chamber. Two-way ANOVA showed a significant effect for factor choice: [test, *F*(2,24) = 55.705; *p* < 0.01; re-test, *F*(2,24) = 54.955; *p* < 0.01]. The day after post-trial infusion, extinction (ext) 1, both groups vehicle and prazosin showed CPP for Amph paired chamber. Two-way ANOVA showed a significant effect for factor choice during ext 1: *F*(2,24) = 47.082; *p* < 0.01, but non-significant interaction treatment × choice: *F*(2,24) = 0.06, ns (**Figures 4A,B**). Prazosin post-trial group reached extinction criterion on the fourteenth trial. Statistical analyses revealed significant effect of choice for both ext 13 [*F*(2,24) = 41.957; *p* < 0.01] and ext 14 [*F*(2,24) = 29.457; *p* < 0.01]. However, Duncan’s test showed that in both days prazosin treated mice did not show difference in time spent in Amph- in comparison with saline-paired chamber (**Figure 4B**). Likewise, vehicle post-trial group reached the extinction criterion on the 15th trial. Two-way ANOVA revealed significant effect of the factor choice in both ext 14 [*F*(2,24) = 29.457; *p* < 0.01] and ext 15 [*F*(2,24) = 23,642; *p* < 0.01]. Duncan’s test showed that in both days vehicle post-trial mice have spent an equal amount of time in the chambers paired with Amph and saline (**Figure 4A**).

**FIGURE 5:**
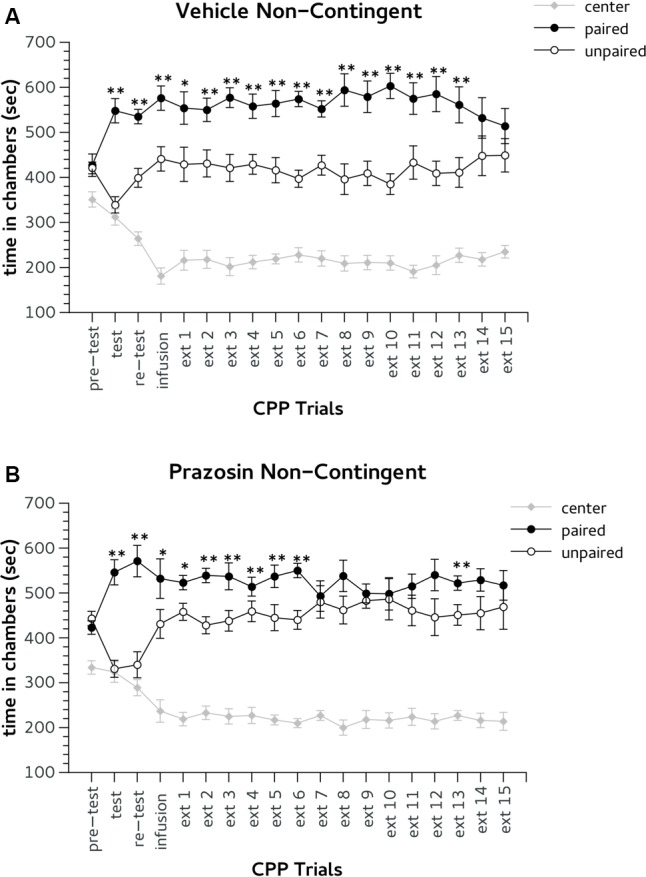
Effects of non-contingent infusion of prazosin in prelimbic (PL) prefrontal cortex on expression and extinction of an acquired conditioned place preference (CPP) induced by systemic injection of 2.5 mg/Kg of amphetamine. In the *x*-axis are days. In the *y*-axis is time spent in center, paired, and unpaired chamber during pre-test, test, re-test, and non-confined extinction trials in animals assigned to vehicle **(A)** and prazosin **(B)**. All data are expressed as mean (second ± SE) time spent in center, paired, and unpaired chambers. ^∗^*p* < 0.05, ^∗∗^*p* < 0.01 in time spent in paired in comparison with unpaired chamber in vehicle and prazosin infused mice.

Finally, we verified whether PL prazosin infusion produced a facilitator effect on the extinction of Amph-induced CPP independently by the exposure to the CPP trial context. In this experiment prazosin or vehicle were administrated 5–6 h before the CPP trial (non-contingent groups). During pre-test mice randomly assigned to non-contingent vehicle (*n* = 8) or prazosin (*n* = 10) groups did not show preference for the lateral chambers (**Figures 5A,B**). Two-way ANOVA revealed non-significant effect for treatment × choice interaction: *F*(2,32) = 0.423, ns. The factor choice was significant: *F*(2,32) = 11.332, *p* < 0.01. Duncan’s *post hoc* test confirmed that both groups spent a greater amount of time in two lateral chambers in comparison with the central alley.

During CPP test and re-test all mice showed the preference for Amph paired chamber. Two-way ANOVA showed a significant effect for factor choice: [test, *F*(2,32) = 40.386, *p* < 0.01; re-test, *F*(2,32) = 42.204, *p* < 0.01]. In the CPP trial after non-contingent infusion both groups showed a preference for the Amph-paired chamber (**Figures 5A,B**). ANOVA revealed no significant effect for treatment × choice interaction: *F* = (2,32) = 0.850, ns; but a significant effect for the factor choice: *F* = (2,32) = 41,252, *p* < 0.01. Non-contingently prazosin infused mice reached the extinction criterion on trial 8. ANOVA revealed a significant effect for the factor choice in both ext 7 [*F* = (2,32) = 47,797, *p* < 0.01] and 8 [*F* = (2,32) = 50,338, *p* < 0.01]. However, Duncan’s test showed that in both days only non-contingent vehicle mice spent more time in the chamber previously paired with (*p* < 0.01) (**Figure 5A**). Note, that non-contingent prazosin mice showed a spontaneous recovery for Amph CPP during extinction trial 13 (**Figure 5B**). Non-contingent vehicle group reached the criterion of two consecutive daily lack of preference on the 15th trial. Two-way ANOVA showed a significant effect of the factor choice in both ext (ext 14 [*F*(2,32) = 34.749, *p* < 0.01] and 15 [*F*(2,32) = 25,623, *p* < 0.01]. Duncan’s test showed that in both days vehicle non-contingent mice spent an equal amount of time in the chambers paired with Amph and vehicle (**Figure 5A**).

### Prelimbic Prazosin Infusion, Immediately before the Exposure to the CPP Trial, Increases BDNF and PSD-95 mRNA Expression in the Nucleus Accumbens

Given the delayed effect of the intra-PL prazosin infusion on the extinction of Amph-induced CPP, we hypothesized that the acute blockade of PL α1-ARs contingent with the exposure to the CPP trial context may induce neuroplastic adaptations in the prefrontal-accumbal network, that underlie the facilitation of extinction. We thus analyzed by quantitative real-time PCR the transcription levels of the neurotrophin *BDNF* and of the synaptic scaffold *PSD-95* as markers of neuroplasticity and synaptic maturation, respectively. Gene expression was measured in punches of IL and PL cortices, and NAc core and shell subregions, of pre-trial prazosin- and vehicle-treated mice. To ensure that both vehicle and prazosin group were equally exposed to extinction trials, mice were sacrificed for mRNA assessment once the preference for Amph CS was extinguished only in prazosin-treated mice, but not necessarily in the vehicle.

**FIGURE 6:**
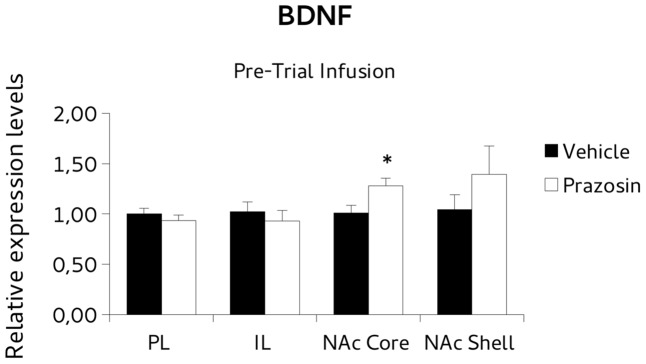
Brain-derived neurotrophic factor (BDNF) mRNA expression in punches of infralimbic (IL) and prelimbic (PL) cortices, and Nucleus Accumbens (NAc) core and shell subregions, measured after the extinction of amphetamine-induced conditioned place preference (CPP), in mice that received pre-trial infusion of prazosin or vehicle in PL. The BDNF mRNA expression level (relative expression levels = fold changes over vehicle, normalized to glyceraldehyde 3-phosphate dehydrogenase (GAPDH) gene) was significantly increased in the NAc core of Prazosin versus Vehicle-infused mice. ^∗^*p* < 0.05.

**FIGURE 7:**
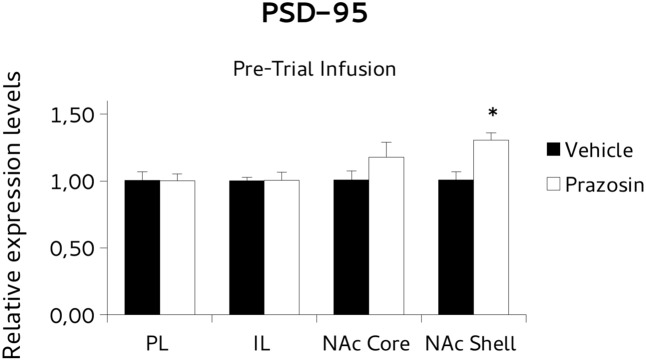
Postsynaptic density protein 95 (PSD-95) mRNA expression in punches of IL and PL cortices, and NAc core and shell subregions, measured after the extinction of amphetamine-induced CPP, in mice that received pre-trial infusion of prazosin or vehicle in PL. The PSD-95 mRNA expression level (relative expression levels = fold changes over vehicle, normalized to GAPDH gene) was significantly increased in the NAc shell of Prazosin versus Vehicle-infused mice. ^∗^*p* < 0.05.

For *BDNF* analysis, paired *t*-test revealed a significant increase of the *BDNF* transcript in NAc core punches of pre-trial PL prazosin-infused mice compared to vehicle [*t*(4) = 3.877, *p* < 0.05]. Non-significant effect was observed in NAc shell [*t*(4) = 0.888, ns], PL [*t*(3) = -0.643, ns] or IL [*t*(4) = -0,697, ns] (**Figure 6**). Similarly, the *PSD-95* mRNA expression level increased significantly in NAc shell punches of pre-trial PL prazosin-infused mice compared to vehicle [*t*(4) = 2.801, *p* < 0.05], while no effect of the drug was observed in NAc core [*t*(3) = 2.315, ns], PL [*t*(3) = -0.197, ns)] or IL [*t*(4) = 0.049, ns] (**Figure 7**). Note that a trend toward an increase was evident in NAc core.

**FIGURE 8:**
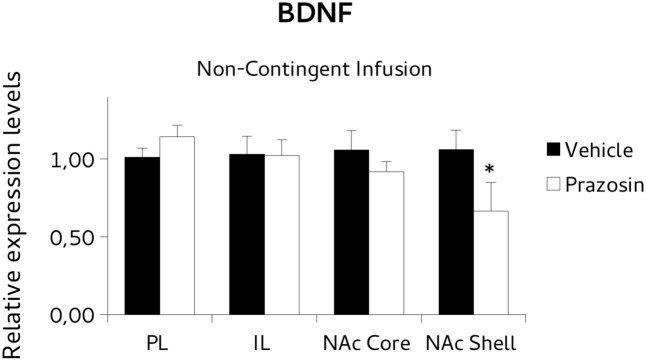
Brain-derived neurotrophic factor (BDNF) mRNA expression in punches of IL and PL cortices, and NAc core and shell subregions, measured after the extinction of amphetamine-induced CPP, in mice that received non-contingent infusion of prazosin or vehicle in PL. The BDNF mRNA expression level (relative expression levels = fold changes over vehicle, normalized to GAPDH gene) was significantly decreased in the NAc shell of Prazosin versus Vehicle-infused mice. ^∗^*p* < 0.05.

**FIGURE 9:**
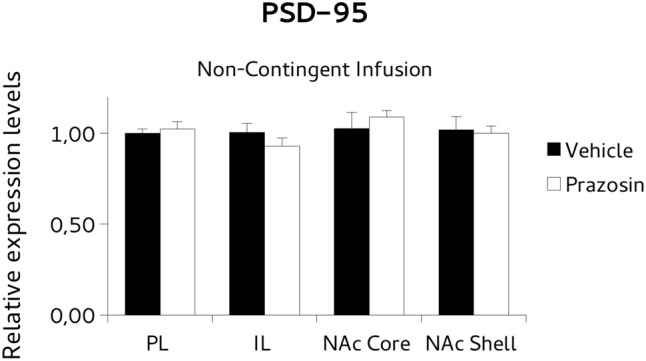
Postsynaptic density protein 95 (PSD-95) mRNA expression in punches of IL and PL cortices, and NAc core and shell subregions, measured after the extinction of amphetamine-induced CPP, in mice that received non-contingent infusion of Prazosin or Vehicle in PL. Non-significant differences were observed in PSD-95 mRNA expression level (relative expression levels = fold changes over vehicle, normalized to GAPDH gene) in either structure.

To test the possibility that the PL prazosin infusion would induce the observed transcriptional modulation independently of the contingent exposure to the CPP trial context, we measured mRNA levels of *BDNF* and *PSD-95* in non-contingently infused prazosin or vehicle mice again when the compartment-preference was extinguished. For *BDNF* analysis, paired *t*-test revealed a significant decrease in the *BDNF* transcript in NAc shell punches of non-contingently PL-infused prazosin mice compared to vehicle [*t*(6) = 2.518, *p* < 0.05). No differences were observed in NAc core [*t*(5) = 1.366, ns], PL [*t*(2) = 0.991, ns], and IL [*t*(2) = -0.087, ns] (**Figure 8**). Non-significant effect of the drug was revealed for the *PSD-95*, in either structure: NAc shell [*t*(6) = 0.661, ns], NAc core [*t*(5) = -1.013, ns], PL [*t*(2) = 1.034, ns], and IL [*t*(1) = -0.332, ns] (**Figure 9**).

## Discussion

This study was aimed at evaluating the role of PL α1-ARs in the maintenance of CPP to Amph. To this aim we assessed the effects of a single pre-reactivation infusion of prazosin in the PL of C57BL/6J mice on the expression of a previously acquired Amph-induced CPP the day of infusion and the following days, when mice were tested drug free. Intra-PL prazosin did not affect expression of Amph-induced CPP the day of infusion, while in subsequent days it produced a clear-cut advance of extinction in comparison with vehicle treated animals. Indeed, from day 5 onward prazosin-treated mice, exposed to the apparatus, showed no preference for the drug-paired chamber differently from vehicle group that extinguished preference for the paired chamber on day 14. Note that this effect of intra-PL prazosin priming is dependent on its infusion before reactivation, since if infused far before or after reactivation it did not affect significantly preference compared with vehicle groups.

A beta-adrenergic receptor antagonist, infused in this area before CPP test, has been reported to block the expression of cocaine-seeking the day of infusion and on the following day when animals are drug-free (Otis et al., 2013). However, in the present study a possible retrieval effect or general memory impairment can be ruled out. Indeed, before the onset of extinction procedure animals assigned to both vehicle and prazosin treatment were able to retrieve drug-associated memory in CPP expressing Amph preference for the paired chamber.

We observed here that no extinction occurred the day of infusion when preference for paired chamber was similar to that shown by vehicle group while preference in treated mice was no more evident after subsequent daily testing sessions. Therefore, a mechanism that could account for not immediately extinction occurring following infusion should have been searched in possible neuroplasticity events triggered by intra-PL prazosin. These could have tagged CS during CPP retrieval, possibly devaluating it or blunting its association with US to produce a process that led to “extinction” of preference later on.

Such neuroplasticity events could have involved not only the brain areas where the antagonist was infused but also other areas that are known to be connected with it and playing a functional role in expression, reconsolidation or extinction of association between CS and drugs.

Lasting efficacy of prazosin in the short period could not be ruled out (Blanc et al., 1994); however, if the compound were effective in testing sessions following intra PL infusion, behavioral effects (extinction) should have been evident also in mice treated non-contingently with exposure to the apparatus. Moreover, post-retrieval treatment did not affect place preference significantly, thus ruling out a possible interference with consolidation of reconsolidation (Dudai and Eisenberg, 2004) produced by intra-PL prazosin prior reactivation due to long-lasting pharmacological action.

To evaluate neuroplastic adaptations we chose to analyze the transcriptional modulation of *BDNF* and *PSD-95 genes.* BDNF is a neurotrophin that is critically involved in the activity-dependent regulation of synaptic structure and function and thus it is considered a reliable marker of neuronal plasticity. BDNF is released upon neuronal activation and exerts its effect on synaptic strength, modulation of dendritic growth, changes in spine density and morphology, by stimulation of protein synthesis and transcription activity, which account for its delayed effect (Carvalho et al., 2008). Interestingly BDNF-induced neuroplasticity has been shown to induce extinction (Rosas-Vidal et al., 2014).

PSD-95 is the most abundant scaffolding protein at mature glutamate synapses and is essential for synaptic maturation and plasticity (El-Husseini et al., 2000). BDNF signaling is known to promote PSD-95 translation and trigger transport of PSD-95 to the synapse where it contributes to the structural and functional maturation of synapses (Yoshii et al., 2011).

In our experiment we assessed transcriptional expression of *BDNF* and *PSD-95* in mice that had extinguished CPP. Specifically, we evaluated mRNA levels in PL and IL cortices and NAc subdivision shell and core (areas of a prefrontal-accumbal network modulating incentive salience and extinction).

The results showed that prazosin-treated mice having extinguished preference showed an increase in *BDNF* mRNA expression in NAc core, and an increase of *PSD-95* in NAc shell, in comparison with vehicle, while non-significant effects on expression of *BDNF* or *PSD95* were evident in PL and IL, or in all punches of animals that were infused with the antagonist non-contingently with reactivation, but in NAc shell where lower *BDNF* levels were evident.

Overall these results indicate the NAc core and shell as key target structures involved in the neuroplastic adaptations mounted by PL cortex upon prazosin infusion and associated with the facilitation of extinction. Interestingly, we found that in NAc core the significative increase in *BDNF* mRNA levels was associated to similar trend in *PSD-95* accordingly to previous reports (Li et al., 2013). This data could be interpreted in light of the different role of the two molecules in neuroplastic mechanisms. While *BDNF* indicates an ongoing neurotrophic activity in neurons, *PSD-95* indicates the morphological result of neuroplasticity, consequence of *BDNF* activity and thus delayed in time. The transcriptional modulation observed demonstrates an active dynamics of neuroplasticity mechanisms induced by PL-infused prazosin in NAc, despite the detection of mRNA rather then protein, allows us only to speculate on the functional direction of the differences observed.

These results, that can not rule out other different neural plasticity effects in cortical functioning in our experimental conditions, indicate that PL (target area of prazosin priming) is likely to drive functional process in subcortical areas that result in neuroplasticity changes strongly related to extinction of Amph-induced CPP.

Indeed, *BDNF* and *PSD95* expression increase was evident only in animals that had extinguished, but not in vehicle matched group or in mice that received prazosin out of the retrieval session, namely non-contingently with exposure with the CPP apparatus and that not extinguished yet preference for Amph paired CS. It worth noting also that these effects were exposure-dependent, a relevant result in the perspective of possible drug assisted behavioral therapy pointed out by our preclinical model.

Moreover behavioral and molecular results are relevant since they point out a possible “priming” effect of the antagonist that suggests a potential therapeutic power (to be further ascertained), given by efficacy of single or sporadic pharmacological (prazosin) treatment with the huge advantage of limiting possible side effects of a repeated treatment.

Note that α1-AR stimulation was reported to induce neuroplasticity (Sawaki et al., 2003; Neverova et al., 2007; McElligott et al., 2010), therefore antagonists should be possibly able to impair neuroplasticity processes induced by receptor activation but is questionable that antagonists themselves produce neuroplastic effects through receptor blockade *per se*.

A body of research of R.S. Duman and his associates has addressed authoritatively the mechanisms of neural plasticity induced by receptor antagonists. They have demonstrated that two antagonists of NMDA or muscarinic receptor, ketamine and scopolamine respectively, provided of clear antidepressant properties, after acute intra-cortical infusion produce increased glutamate transmission that is able to trigger neuroplasticity mechanisms leading to long-lasting behavioral and synaptic changes, also through BDNF. The action of acute (single) administration is transient, and its effects on glutamate depend on blockade of GABA interneurons tonic firing (Duman and Aghajanian, 2012, 2014; Wohleb et al., 2017). Moreover, it has been recently reported that acute ketamine infused in mPFC modulate extinction of conditioned fear (Girgenti et al., 2017).

In these studies neuroplasticity effects occur in cortical areas where drugs are infused, while our present results show neuroplasticity occurring in NAc, although other neural plasticity mechanisms not assessed here cannot be ruled-out. These effects on NAc are consistent with a role of mesolimbic system in modulation of salient stimuli and motivated behavior characterizing CPP to a psychostimulant such that used in our experiments. However, neural plasticity events are likely to originate in the prefrontal cortex and need the animal to be exposed to CS, as is clearly indicated by the results of prazosin infusion non-contingently to the extinction process.

Similarly to what shown for ketamine and scopolamine, we hypothesize that prazosin triggers mechanisms that involves different neurotransmitters. This view is supported by unpublished results obtained in reverse microdialysis experiments showing that prazosin, in CPP extinction procedure, increases dopamine outflow while decreases GABA levels in PL. These effects could spur molecular events, possibly involving glutamate, to promote neural and behavioral plasticity. It is worth noting to mind that a possible increase of prefrontal cortical dopamine by prazosin was hypothesized by Blanc and coworkers a couple of decades ago (Blanc et al., 1994).

Thus it is conceivable that prazosin, possibly altering neural events involving different neurotransmitters in close neurons in PL or in distal neuron of areas connected with PL (e.g., NAc), directly or through trans-neuronal feedback, is able to trigger neuroplastic adaptations such those we observed here. Further study could possibly elucidate this point.

Present results can involve the action of different factors. NE transmission in prefrontal cortex is pivotal in acquisition of motivational properties of different classes of rewarding and aversive stimuli that spur NE release in this area (Ventura et al., 2005, 2007, 2008). Moreover, exposure to appetitive CS can induce prefrontal NE release and the magnitude of this release is positively related to the salience of the US during the training session (Mingote et al., 2004; Ventura et al., 2008). Note that increased prefrontal NE release leads to increase of DA release in the NAc and the paired prefrontal-accumbal catecholamine are crucial in attribution of motivational salience involving highly salient USs (Darracq et al., 1998; Ventura et al., 2003, 2005, 2007).

Therefore, we hypothesize that repeated exposure to the environment paired with Amph was able to increase NE levels in mPFC in control (vehicle) animals, and that this increase contributed to maintain the motivational properties of CS fostering drug-seeking behavior. Conversely, acute intra-PL prazosin blocked the effects of NE release on α1-ARs in animals exposed to Amph conditioned environment. This resulted in blunting the motivational properties of CS and reducing the persistence of CPP in following days, supported by neuroplasticity processes as those observed in the NAc. Moreover, it is well known that repeated non-reinforced exposure to the CS reduces the CR leading to its extinction (Pavlov, 1927; Berman et al., 2003). Accordingly, extinction is conceptualized as a new context-dependent learning that competes with the original learning to control motivation and behavior (Bouton, 2004; McNally, 2014). Therefore, during extinction trials, the original association (Amph-CS) could have undergone a substantial reduction of salience under prazosin action, due to the antagonist-induced impairment of prelimbic NE transmission following exposure to the CS.

Our results point to neuroplasticity effects in NAc, suggesting a prefrontal-accumbal modulation of CS related motivational salience. These effects may be due to prazosin blockade of activation of postsynaptic Gq-coupled α1-ARs and PKC that have been shown to inhibit persistent activity of prefrontal pyramidal neurons (Birnbaum et al., 2004), Therefore, it can be hypothesized that prazosin in PL increases the activity of pyramidal neurons in this area counteracting the hypoactivity of mPFC possibly induced by both Amph administration and conditioned NE release and thus sustaining the inhibitory control over drug-seeking, a view that needs further study to be ascertained.

In addition to this, a possible interplay between PL and IL should be taken into account. Indeed, we have recently shown that NE transmission modulates extinction of Amph-induced CPP through a balance between PL and IL (Latagliata et al., 2016). Therefore, exposure to Amph-paired environment in intra-PL prazosin animals could have shifted the weight of NE in favor of the IL, thus fostering the emergence of extinction trace that would prevail in modulating behavioral outcome. Further experiments could elucidate this point. However, it is worth noting that we observed here changes in BDNF and PSD-95 expression in both accumbal subregions core and shell that are well known to be involved in motivation and in extinction also due to their connections with PL and IL (Bouton, 2004; Miller and Marshall, 2004; Kalivas et al., 2005; Peters et al., 2009; Martín-García et al., 2014; McNally, 2014). Thus, it may be that prefrontal driven modulation of NAc core and shell, resulting in *BDNF* and *PSD-95* expression, promote extinction of US-CS association and/or blunt motivational salience of CS respectively.

Our present results leave a number of points open. Additional specific molecular mechanisms involved in neuroplasticity could be further investigated, to ascertain the effects of acute prazosin in short- and in long-term plasticity in PL. The involvement of other neurotransmitter action, such as dopamine, GABA, glutamate on plasticity and behavior must be assessed. Unveiling the role of different neurotransmitters can help to envisage possible acute therapy based on combined treatment with compounds acting synergistically on different neural mechanisms to spur synaptic and learning processes, possibly at low dosage to minimize side effects. The effects of acute systemic antagonist administration on brain and behavior are to be elucidated and compared with those of intra-cerebral infusion, to assess its therapeutic power.

## Conclusion

A single pre-reactivation infusion of prazosin in the PL of C57BL/6J mice did not affect expression of Amph-induced CPP the day of infusion, while in subsequent days it produced a clear-cut advance of extinction, an effect depending on contingent exposure to retrieval, since if infused far from or after reactivation it did not affect preference.

*BDNF* and *PSD-95* expression increase in the NAc Core and Shell, that are part of a prefrontal-accumbal network modulating incentive salience and extinction, was evident only in animals that had extinguished, but not in vehicle matched group or in mice that had received prazosin out of the retrieval session. These neuroplasticity events triggered by intra-PL prazosin could have tagged CS during CPP retrieval, possibly devaluating it or blunting its association with US, point to a mechanism that could account for non-immediately expressed extinction.

Both behavioral and molecular results are relevant since they point, for the first time to our knowledge, to a possible “priming” effect of prazosin that suggests a potential therapeutic power (to be further ascertained) of the antagonist for maladaptive memories, resulting by the efficacy of a single or sporadic pharmacological treatment allowing to avoid possible side effects of repeated drug treatment.

## Author Contributions

EL and SP-A designed research; EL, GC, and MS performed behavioral experiments; EL, LL, VO, and AR performed RT-PCR; EL, GC, LL, and SP-A analyzed data; EL, LL, and SP-A wrote the paper.

## Conflict of Interest Statement

The authors declare that the research was conducted in the absence of any commercial or financial relationships that could be construed as a potential conflict of interest.

## Abbreviations

α1-ARs: alpha1-adrenergic receptors
Amph: amphetamine sulfate
BDNF: brain-derived neurotrophic factor
CPP: conditioned place preference
IL: infralimbic mpFC
mpFC: medial prefrontal cortex
NAc: nucleus accumbens
PL: prelimbic mpFC
PSD-95: post-synaptic density 95.
